# Effect of Preactivation on Torque Enhancement by the Stretch-Shortening Cycle in Knee Extensors

**DOI:** 10.1371/journal.pone.0159058

**Published:** 2016-07-14

**Authors:** Atsuki Fukutani, Jun Misaki, Tadao Isaka

**Affiliations:** 1 Research Organization of Science and Technology, Ritsumeikan University, Kusatsu, Shiga, Japan; 2 Japan Society for the Promotion of Science, Research Fellowship for Young Scientists, Chiyoda-ku, Tokyo, Japan; 3 Graduate School of Sport and Health Science, Ritsumeikan University, Kusatsu, Shiga, Japan; 4 Faculty of Sport and Health Science, Ritsumeikan University, Kusatsu, Shiga, Japan; University of Debrecen, HUNGARY

## Abstract

The stretch-shortening cycle is one of the most interesting topics in the field of sport sciences, because the performance of human movement is enhanced by the stretch-shortening cycle (eccentric contraction). The purpose of the present study was to examine whether the influence of preactivation on the torque enhancement by stretch-shortening cycle in knee extensors. Twelve men participated in this study. The following three conditions were conducted for knee extensors: (1) concentric contraction without preactivation (CON), (2) concentric contraction with eccentric preactivation (ECC), and (3) concentric contraction with isometric preactivation (ISO). Muscle contractions were evoked by electrical stimulation to discard the influence of neural activity. The range of motion of the knee joint was set from 80 to 140 degrees (full extension = 180 degrees). Angular velocities of the concentric and eccentric contractions were set at 180 and 90 degrees/s, respectively. In the concentric contraction phase, joint torques were recorded at 85, 95, and 105 degrees, and they were compared among the three conditions. In the early phase (85 degrees) of concentric contraction, the joint torque was larger in the ECC and ISO conditions than in the CON condition. However, these clear differences disappeared in the later phase (105 degrees) of concentric contraction. The results showed that joint torque was clearly different among the three conditions in the early phase whereas this difference disappeared in the later phase. Thus, preactivation, which is prominent in the early phase of contractions, plays an important role in torque enhancement by the stretch-shortening cycle in knee extensors.

## Introduction

Muscle force is enhanced by countermovement. This phenomenon is called the stretch-shortening cycle (SSC). The proposed mechanism of force enhancement by countermovement is as follows: stretch reflex [1, 2], tendon elongation [3, 4] preactivation [5, 6] and residual force enhancement [7, 8].

Many studies, including animal and human experiments have been conducted to elucidate the mechanism of SSC. For example, Bobbert et al. [9] compared the jump height obtained between the countermovement jump (SSC) and squat jump (non-SSC), and they reported that the jump height was higher for the countermovement jump than for the squat jump. They indicated that force enhancement by SSC would be mainly caused by a higher joint torque in the early phase of push-off, which was attributable to preactivation, and the influence of the stretch reflex, tendon elongation, and residual force enhancement would be small. On the other hand, Kawakami et al. [10] suggested that tendon elongation contributes to the enhanced performance of plantar flexion by SSC, because tendon elongation causes appropriate fascicle behavior that produces muscle force (i.e., muscle-tendon interaction), and tendon elongation results in storing and releasing elastic energy. Considering these conflicting results, the mechanism of SSC should be re-examined.

To elucidate the precise mechanism underlying SSC, the controlling activation level is important because if the activation level between SSC and non-SSC trials is not identical, it is difficult to fairly compare the jump height and/or ground reaction force. Likewise, kinematic variables such as the range of motion and angular velocity should also be controlled. Considering these points, it is challenging to examine the precise mechanism underlying SSC in dynamic multi joint movements with voluntary effort. To overcome these problems, Fukutani et al. [11] adopted an electrically-evoked single joint movement. In this study, the range of motion and angular velocity were controlled between trials, and an equal activation level maintained by artificial electrical stimulation. Based on the data obtained from this experimental setting, they suggested that preactivation is the principal factor of torque enhancement by SSC. However, this suggestion was based on data obtained from plantar flexions, and the physiological characteristics of each joint are not necessarily identical. For example, plantar flexors would have a longer free tendon than knee extensors. In addition, the operating range of muscle fibers (i.e., ascending limb, plateau, or descending limb) would be different between the plantar flexors and knee extensors [12, 13, 14], which is associated with the magnitude of residual force enhancement [7]. Thus, another joint should be examined to obtain more generalized knowledge on this topic. Therefore, the present study compared joint torque in SSC and non-SSC trials to elucidate the mechanism of force enhancement by SSC in knee extensors. We hypothesized that preactivation would mainly contribute to torque enhancement by SSC in knee extensors, which is similar to that reported in previous studies [9, 11].

## Materials and Methods

### Subjects

Twelve recreationally-trained healthy young men (mean ± standard deviation, 23.6 ± 2.1 years; height, 1.69 ± 0.02 m; body mass, 65.3 ± 6.5 kg) voluntarily participated in the present study. The purpose and risks of this study were explained to each volunteer, and written informed consent was obtained from all subjects. The Ethics Committee on Human Research of Ritsumeikan University approved this study (IRB-2014-026).

### General experimental setup

In this study, a dynamometer (Biodex; SAKAImed, Tokyo, Japan) was used to test the motion of the knee extensions. The following three conditions were tested: (1) concentric contraction without preactivation (CON), (2) concentric contraction following eccentric preactivation (ECC), and (3) concentric contraction following isometric preactivation (ISO). All muscle contractions were evoked by electrical stimulation (SEN-3401; Nihon Kohden, Tokyo, Japan). Ultrasonographic measurements (SSD-3500; Aloka, Tokyo, Japan) were performed simultaneously during the aforementioned three conditions to examine the behavior of the fascicle, i.e., fascicle length and pennation angle of the vastus lateralis.

### Motion control and torque measurement

**Fig 1:**
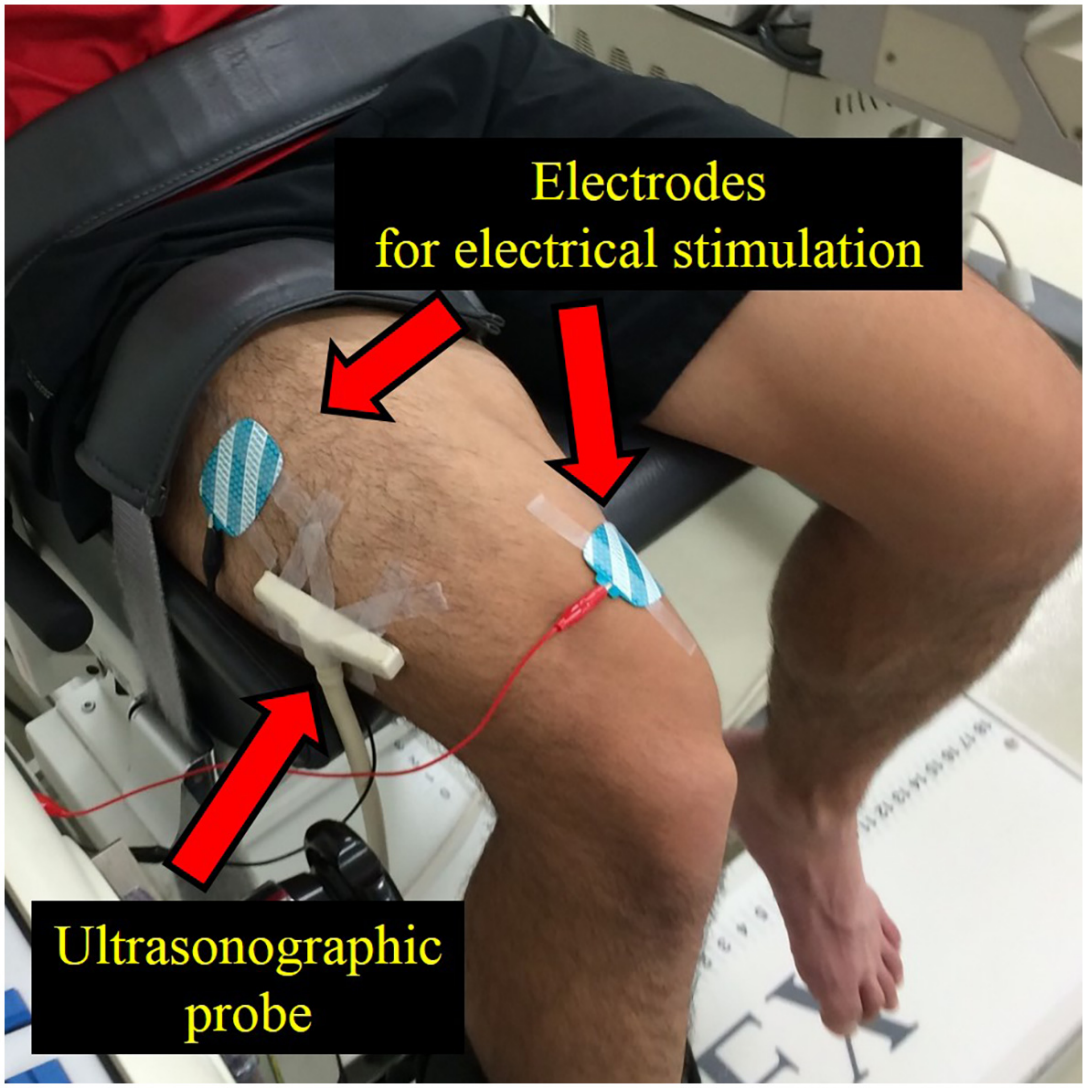
Schematic illustration of the experimental settings.

**Fig 2:**
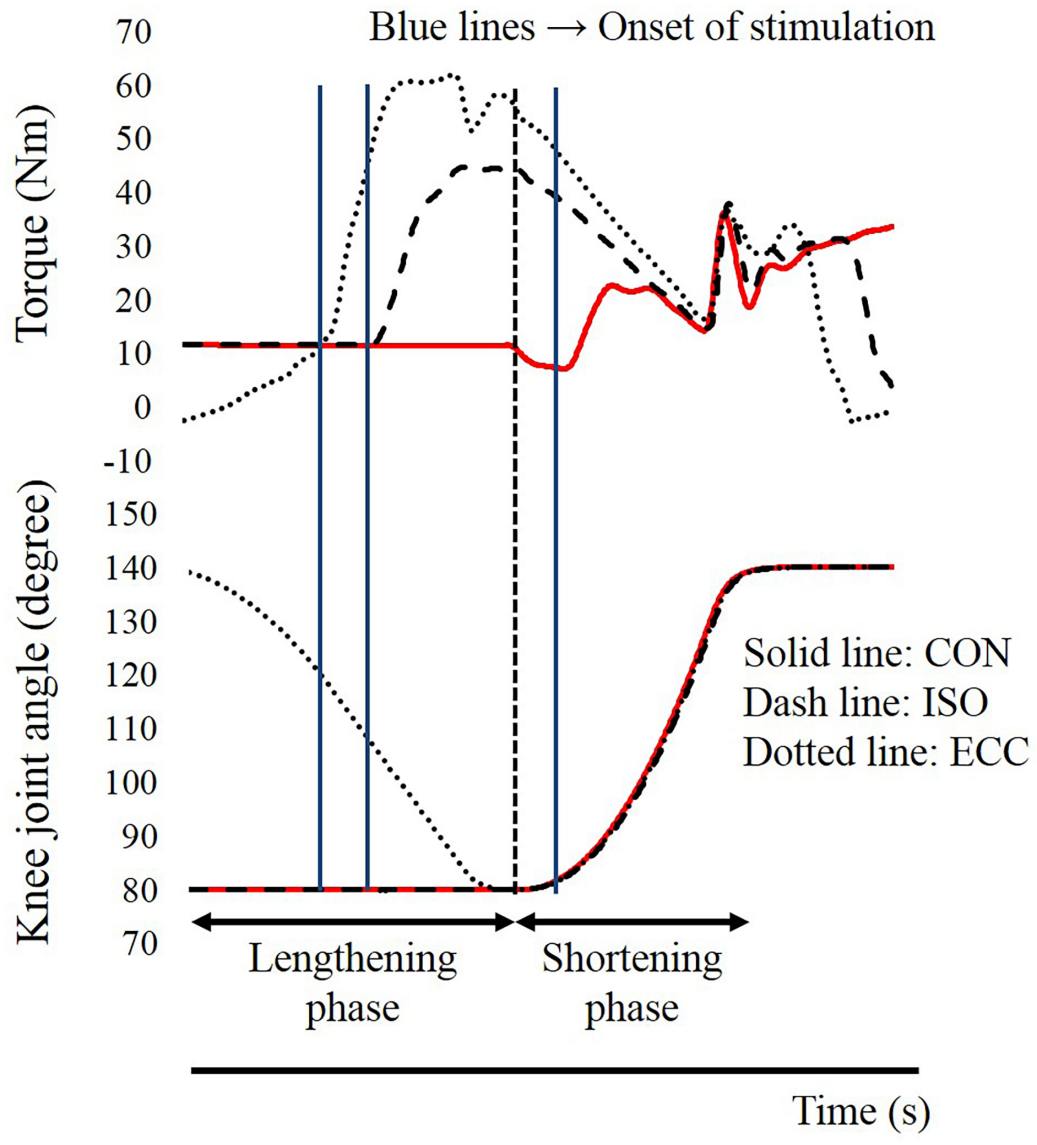
Time course changes in the torque and knee joint angles. CON: concentric contraction without preactivation. ISO: concentric contraction with prior isometric contraction. ECC concentric contraction with prior eccentric contraction.

In this study, knee extension of the right leg was performed. The hip joint angle was fixed at 70 degrees flexion (anatomical position: 0 degrees) throughout the experiment. The upper body and right thigh were fixed on the dynamometer by straps. The knee joint angle was adjusted by the dynamometer. The range of motion of the dynamometer was set from 140 to 80 degrees (full extension: 180 degrees). The joint angular velocities of the shortening phase (i.e., from 80 to 140 degrees) and the lengthening phase (from 140 to 80 degrees) were set at 180 degrees/s and 90 degrees/s, respectively. To evoke muscle contractions, two electrodes (4 × 5 cm) were placed on the muscle belly of the vastus medialis and the vastus lateralis (Fig 1). Before the electrodes were placed, the areas were shaved and cleaned with alcohol. Electrical stimulation was applied with the following parameters: pulse frequency, 100 Hz; pulse duration, 0.5 ms; train duration, 1.3 s. The intensity of electrical stimulation was modulated to evoke 25% of the peak torque attained during maximal voluntary isometric contraction in the knee extensors with the knee joint angle at 90 degrees. In concrete, the maximal voluntary isometric contraction was performed, and the peak joint torque recorded in this contraction was set at 100% intensity. Next, the intensity of electrical stimulation was adjusted to evoke 25% of the 100% intensity at the corresponding joint angle. This electrical stimulation intensity was applied to all contractions. All the contractions mentioned hereafter were electrically-evoked contractions, not voluntary contractions. Joint torque, joint angle, and the timing of electrical stimulation used during each condition are shown in Fig 2. Each condition was conducted one time. Because all the contractions were conducted by electrical stimulation, instruction to the subjects was “As relax as possible” to avoid redundant voluntary muscle activation. In the CON condition, to evoke concentric contraction without preactivation, electrical stimulation was applied when the knee joint angle passed 82 degrees in the shortening phase (i.e., from 80 to 140 degrees). Thus, this condition did not include isometric or eccentric contractions before the concentric contraction. In the ECC condition, electrical stimulation was applied when the knee joint angle passed 120 degrees in the lengthening phase (i.e., from 140 to 80 degrees). Thus, this condition included eccentric contraction just before the concentric contraction, and range of eccentric contraction phase was from 120 to 80 degrees. In the ISO condition, which was different from the other two conditions, the position of the dynamometer was maintained at 80 degrees before shortening phase. The timing of electrical stimulation was 0.5 s before the dynamometer was moved from 80 to 140 degrees (i.e., shortening phases). As a result, this condition included isometric contraction just before the concentric contraction. The duration of isometric contraction was 0.5 s. Thus, both the ECC and ISO conditions included preactivation, but the ISO condition did not include eccentric contraction, which is essential to evoke residual force enhancement [7, 8]. These three conditions were performed randomly with more than two-minute intervals between the trials. Joint torques recorded at 85 degrees (the early phase of concentric contraction), 95 degrees, and 105 degrees (the later phase of concentric contraction) were used in the following analyses. The joint torque and joint angle were recorded with a sampling frequency of 4,000 Hz (Power lab 16/30; ADInstruments, Bella Vista, Australia). Joint torques recorded at 85, 95, and 105 degrees were compared among the CON, ECC, and ISO conditions.

### Ultrasonographic measurement

**Fig 3:**
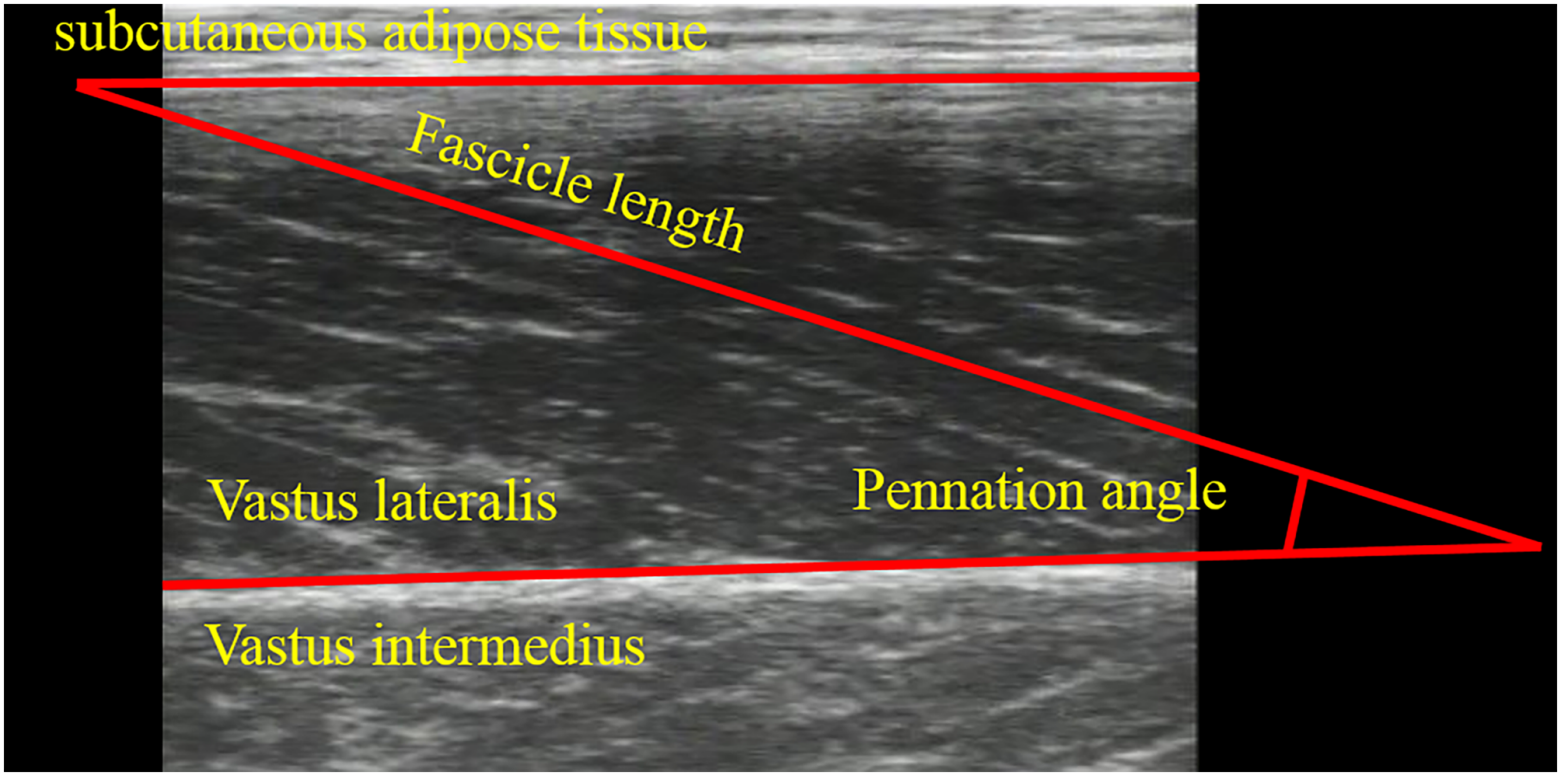
Ultrasonographic image of the vastus lateralis.

Ultrasonography with a linear array probe (UST-5710; Aloka, Tokyo, Japan) was used to obtain images of the muscle belly of the vastus lateralis (Fig 1), which is one of the knee extensors. The fascicle length and pennation angle were obtained at 85, 95, and 105 degrees, which corresponded to the joint torque measurements. The fascicle length was defined as the distance between the intersection composed of the superficial aponeurosis and fascicle, and the intersection composed of the deep aponeurosis and fascicle (Fig 3). The pennation angle was defined as the internal angle composed of the fascicle and deep aponeurosis (Fig 3). When the entire fascicle was not visible, the linear extrapolation method, which has been widely used [15, 16, 17], was adopted to calculate the fascicle length. The sampling frequency of ultrasonography was set at 30 Hz. Acquired images were analyzed using Image J 1.47v software (National Institutes of Health, Bethesda, MD, US). The fascicle length and pennation angle were compared among the three conditions at three joint angles. In addition, the magnitude of fascicle shortening was calculated at the following three intervals: (a) from 85 to 105 degrees (full range), (b) from 85 to 95 degrees (the first half), and (c) from 95 to 105 degrees (the latter half). In our previous studies (Fukutani et al, 2015), coefficients of variation and intra-class correlations of joint torque, fascicle length, and pennation angle obtained in the similar experimental condition were calculated. As a result, coefficients of variation were 2.3%, 1.1%, and 1.6%, respectively, and intra-class correlations were 0.995, 0.993, and 0.989, respectively.

### Statistical analyses

Two-way analysis of variance (ANOVA) with repeated measures was used to analyze the interaction (condition × joint angle) and main effect (condition and joint angle) of the joint torque, pennation angle, and fascicle length. If the interaction was significant, one-way ANOVA with repeated measures followed by post-hoc testing (i.e., Bonferroni’s correction) was conducted. To determine the magnitude of fascicle shortening, one-way ANOVA with repeated measure was adopted to examine the main effect (i.e., the condition). The effect size for ANOVA was calculated as the partial *η*^2^. Statistical analyses were performed using SPSS version 20 software (IBM, Tokyo, Japan), with the level of statistical significance set at *P* < 0.05.

## Results

**Fig 4:**
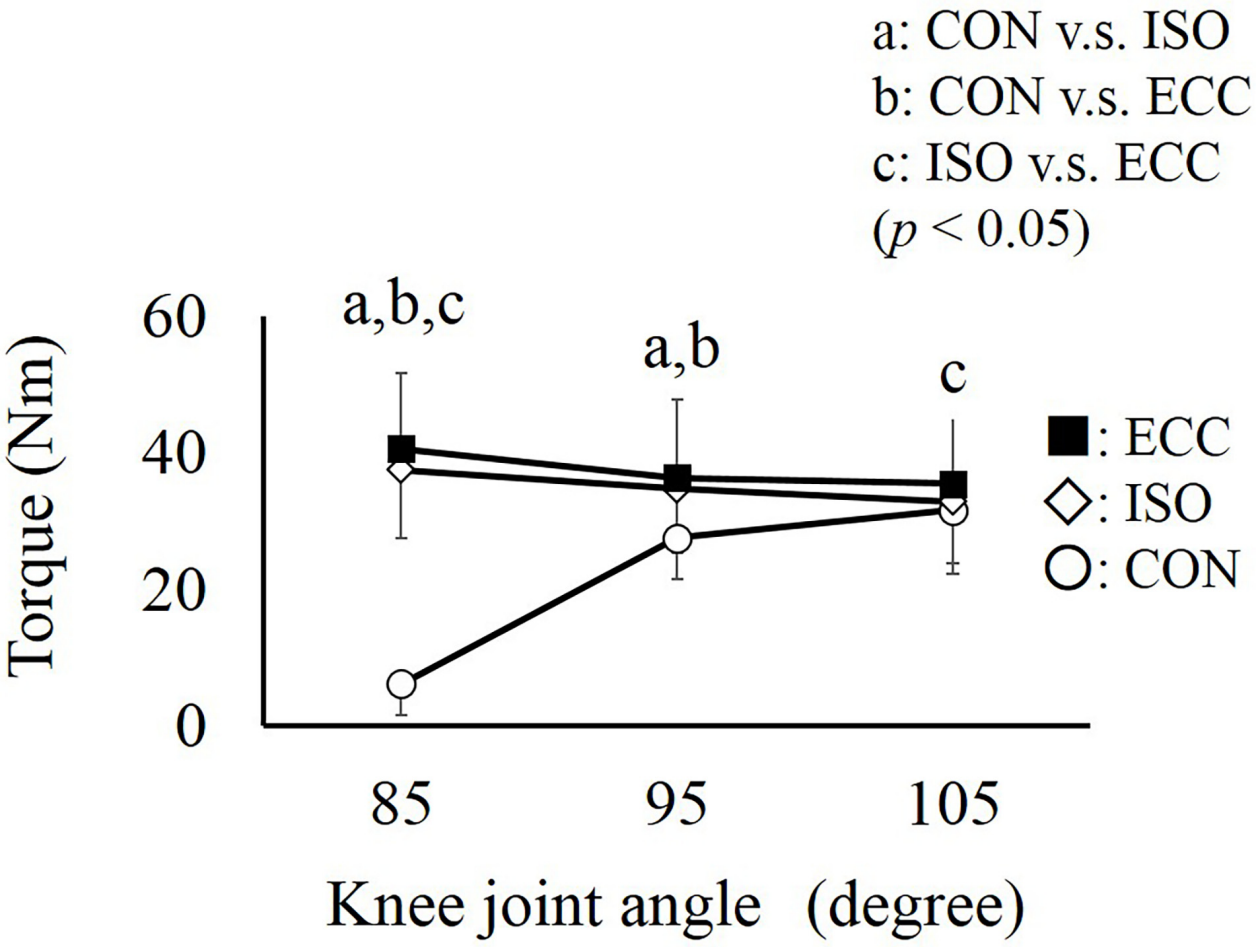
Torque obtained at three knee joint angles. a, b, and c represent significant differences between the CON and ISO, CON and ECC, and ISO and ECC conditions, respectively (*P* < 0.05). Values are presented as mean ± standard deviation. CON: concentric contraction without preactivation. ISO: concentric contraction with prior isometric contraction. ECC concentric contraction with prior eccentric contraction.

Regarding joint torque, two-way ANOVA with repeated measures showed a significant interaction (partial *η*^2^ = 0.942, *P* < 0.001). Subsequent analyses showed that joint torque was significantly larger in the ECC and ISO conditions than in the CON condition at 85 degrees (the early phase). However, these differences among the conditions almost disappeared at 105 degrees (the later phase) (Fig 4).

**Fig 5:**
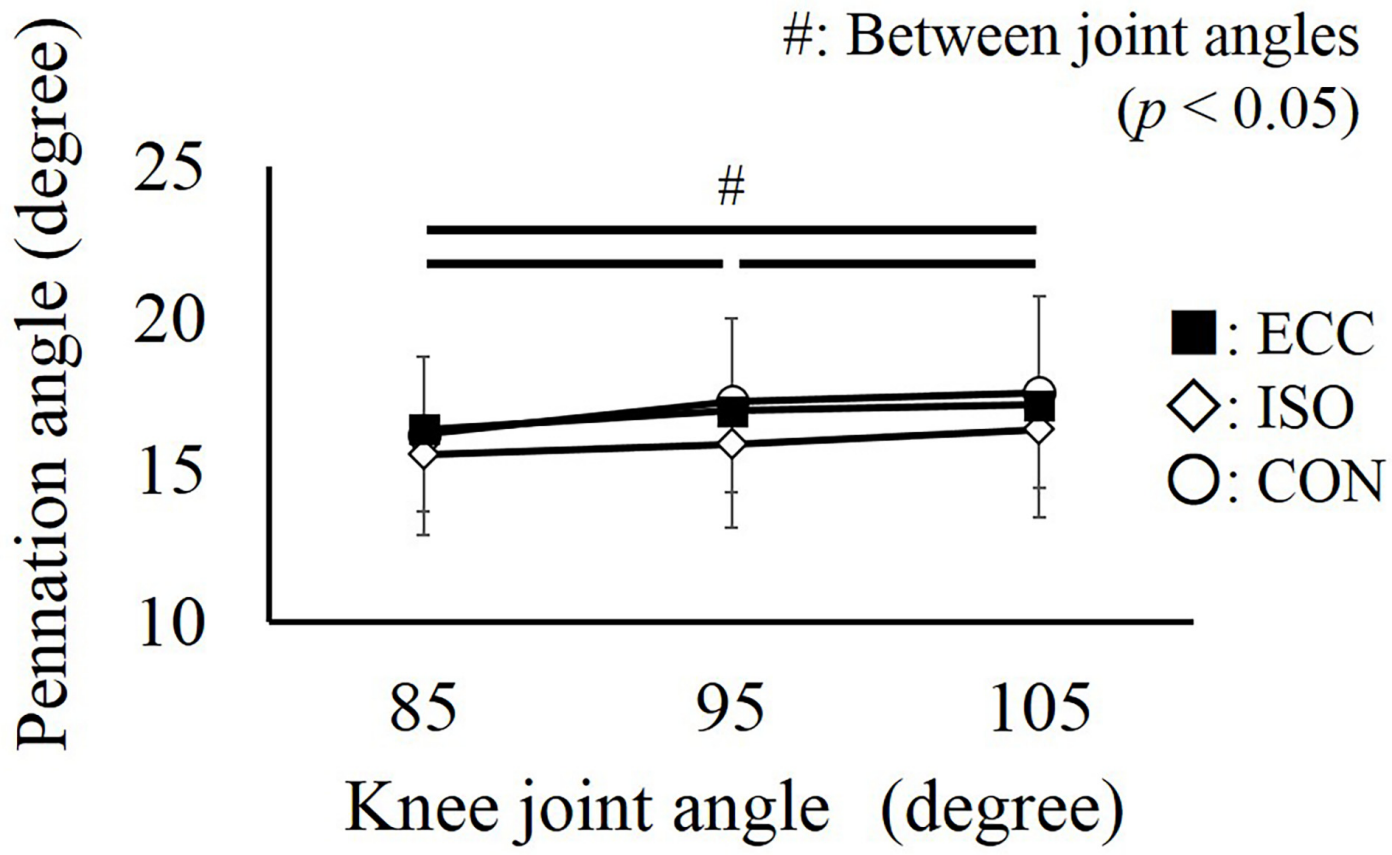
Pennation angle obtained at three knee joint angles. # represents the significant difference among the knee joint angles (*P* < 0.05). No significant differences were found among the CON, ISO, and ECC conditions (*P* > 0.05). Values are presented as mean ± standard deviation. CON: concentric contraction without preactivation. ISO: concentric contraction with prior isometric contraction. ECC concentric contraction with prior eccentric contraction.

Concerning the pennation angle, no significant interaction was found (partial *η*^2^ = 0.067, *P* = 0.633), and a main effect was only found for the joint angle (partial *η*^2^ = 0.526, *P* < 0.001). The pennation angle increased significantly as the knee joint was extended (*P* < 0.001) (Fig 5).

**Fig 6:**
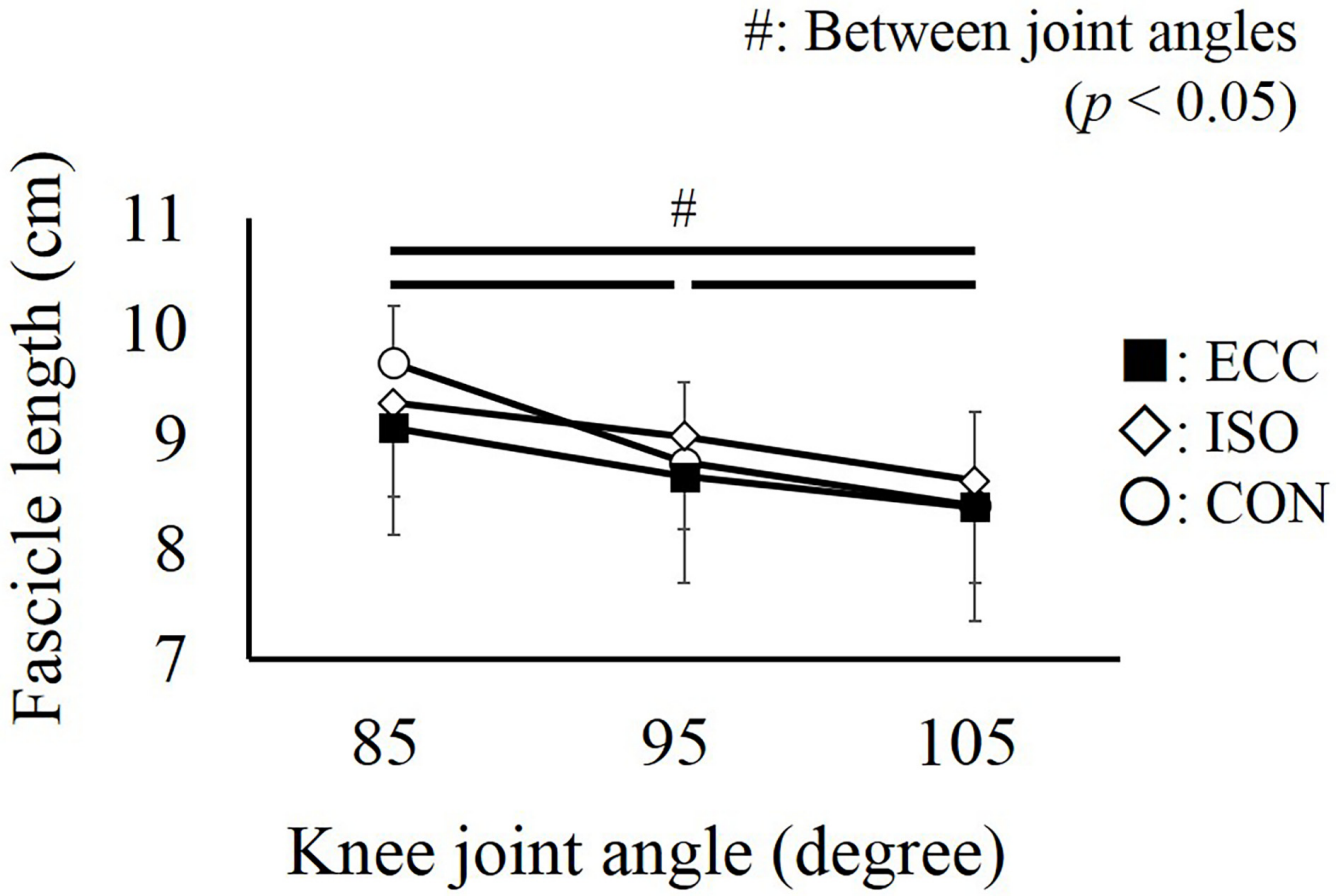
Fascicle length obtained at three knee joint angles. # represents the significant difference among the knee joint angles (*P* < 0.05). No significant differences were found among the CON, ISO, and ECC conditions (*P* > 0.05). Values are presented as mean ± standard deviation. CON: concentric contraction without preactivation. ISO: concentric contraction with prior isometric contraction. ECC concentric contraction with prior eccentric contraction.

Regarding the fascicle length, a significant interaction was found (partial *η*^2^ = 0.471, *P* = 0.001). However, additional analyses showed that the fascicle length was not different among conditions at each joint angle (*P* = 0.089 –*P* > 0.999). On the other hand, fascicle length decreased significantly as the knee joint was extended (*P* < 0.001 –*P* = 0.013) (Fig 6).

**Fig 7:**
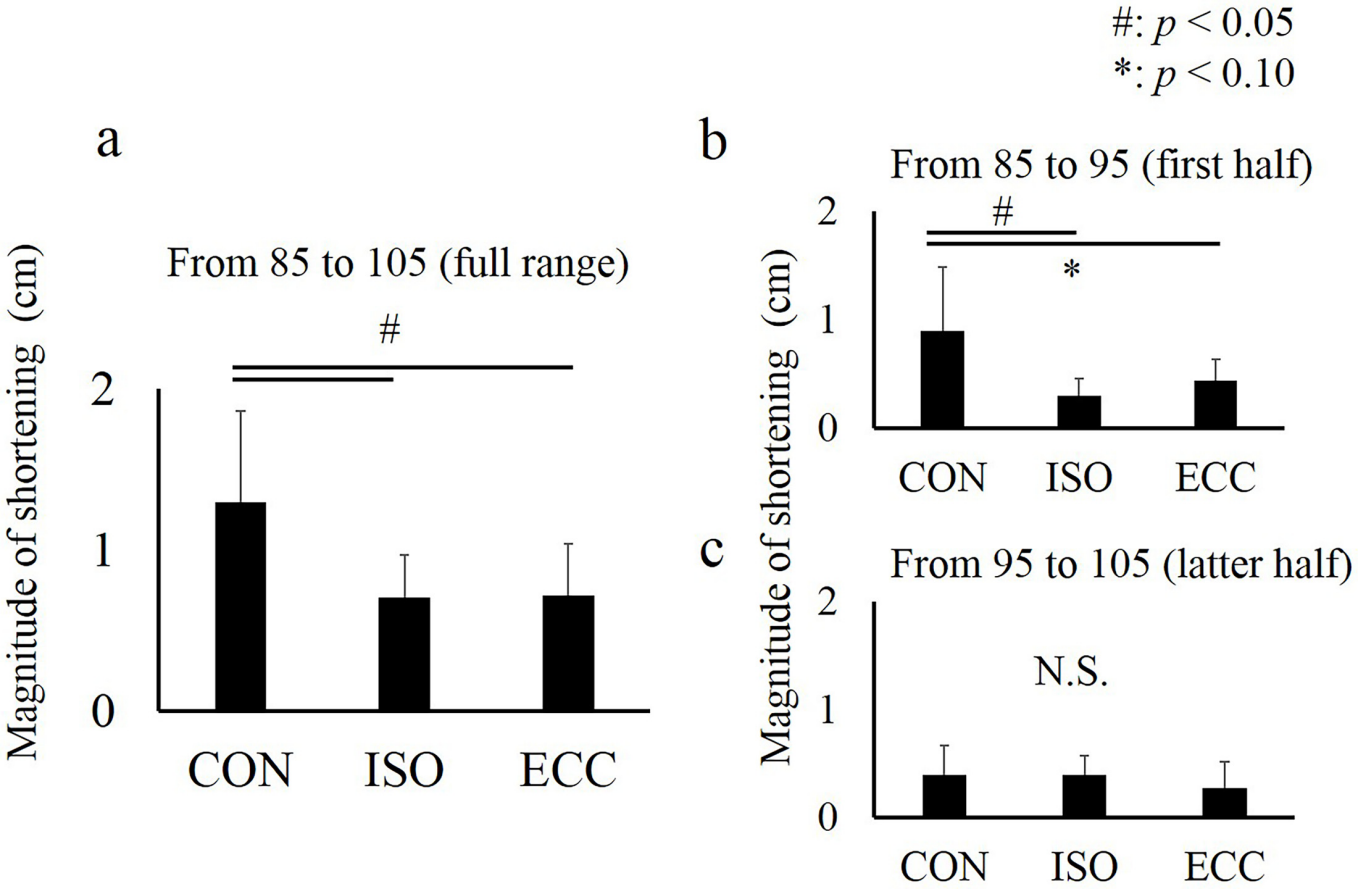
Magnitude of fascicle shortening in the CON, ISO, and ECC conditions at the following intervals: (a) from 85 to 105 degrees (full range), (b) from 85 to 95 degrees (the first half), and (c) from 95 to 105 degrees (the latter half). # represents the significant difference among the CON, ISO, and ECC conditions (*P* < 0.05). * represents significant difference between the CON and ECC conditions (*P* < 0.05). Values are presented as mean ± standard deviation. CON: concentric contraction without preactivation. ISO: concentric contraction with prior isometric contraction. ECC concentric contraction with prior eccentric contraction.

Regarding the magnitude of fascicle shortening, one-way analysis indicated that the magnitude of fascicle shortening calculated by 85 to 105 degrees (full range) and 85 to 95 degrees (the first half) was larger in the CON condition than in the ISO and ECC conditions whereas no significant difference was observed between the ISO and ECC conditions. On the other hand, no significant difference was observed for the magnitude of fascicle shortening calculated by 95 to 105 degrees (the latter half) (Fig 7).

## Discussion

The purpose of this study was to examine the mechanism of torque enhancement by SSC in knee extensors. Compared to the CON condition (i.e., non-SSC condition), joint torque was larger in the ECC and ISO conditions in the early phase of concentric contraction (85 degrees). However, these differences almost disappeared in the latter phase of concentric contraction (Fig 4). Considering the facts that substantial torque enhancement was confirmed even in the ISO condition which did not include eccentric contraction, and that the magnitude of torque enhancement was almost similar between the ISO and ECC conditions (Fig 4), torque enhancement by SSC (i.e., torque enhancement in the ECC condition) would be mainly caused by preactivation.

**Fig 8:**
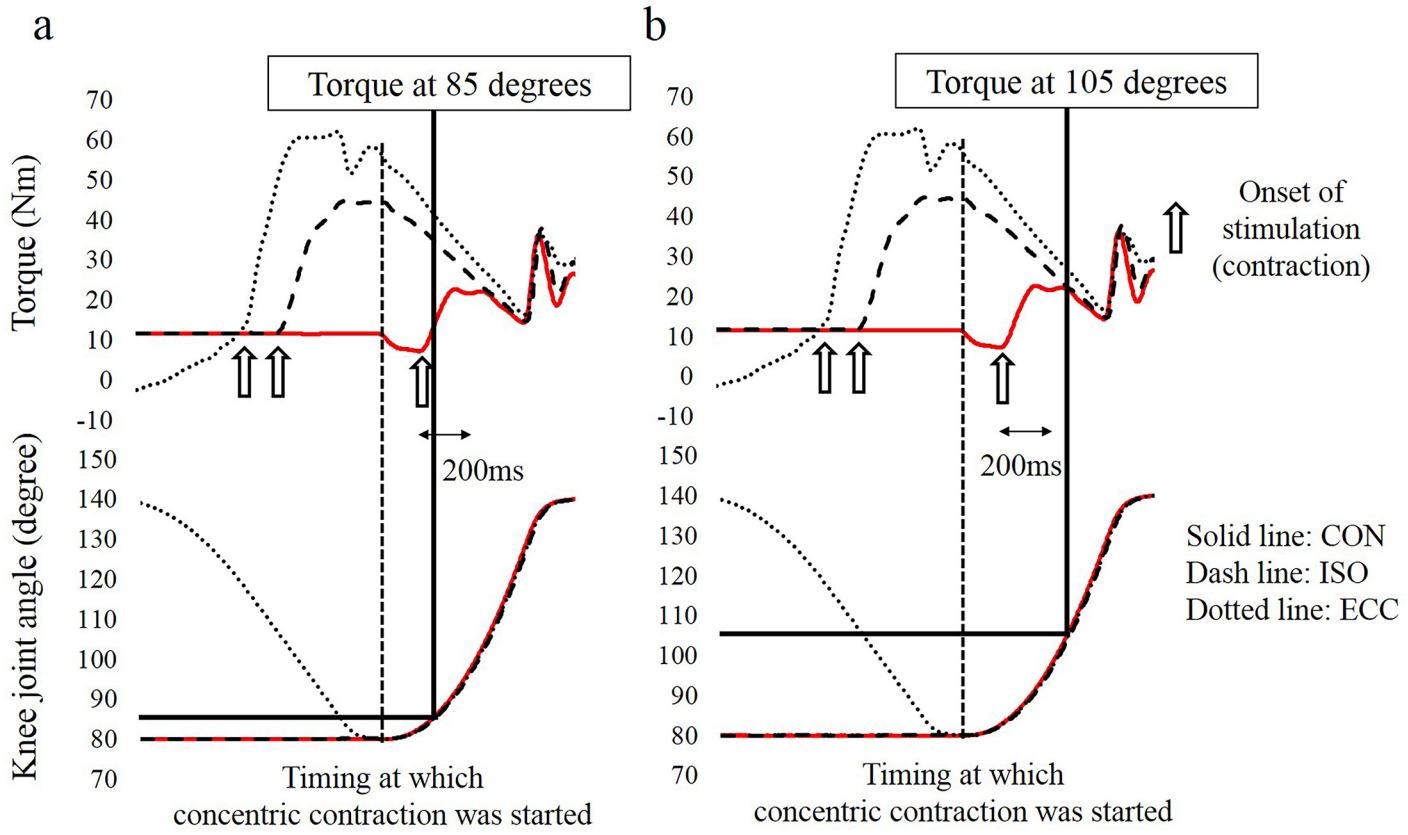
Time course changes in the torque and knee joint angle. Figure 8a shows the timing at which the knee joint reached 85 degrees. Figure 8b shows the timing at which the knee joint reached 105 degrees. Note that the time to increase the joint torque was shorter in 8a (under 30 ms) than in the 8b (over 200 ms). Consequently, torque was still increasing (it did not reach the maximal level) when the knee joint angle reached 85 degrees.

Our result that the larger difference among conditions was observed at 85 degrees (the early phase) whereas this difference disappeared at 105 degrees (the latter phase) can be well explained by the influence of preactivation. Because it takes several seconds to develop joint torque from 0 to 100% (i.e., from a relaxed state to the maximal intensity contraction), joint torque does not reach the maximal level, especially in the early phase of contractions [18]. This delay can be avoided by preactivation. Considering this mechanism, the influence of preactivation is prominent in the early phase of a contraction, and it is negligible in the latter phase of a contraction because joint torque can reach the maximal level even without preactivation due to the long contraction duration. We confirmed that the magnitude of torque enhancement in the ISO and ECC conditions was larger at 85 degrees (Fig 8a). This should be because it only took about 30 ms from the onset of concentric contraction. On the other hand, it took over 200 ms when the knee joint passed 105 degrees; consequently, torque enhancement almost disappeared (Fig 8b). Thus, it is reasonable to speculate that the observed torque enhancement was caused mainly by preactivation.

A slight but significant difference in joint torque was also found not only between the CON and the others conditions (i.e., ISO and ECC), but also between the ISO and ECC conditions, both of which included preactivation. This difference can be explained by the following two reasons: a higher joint torque at the onset of concentric contraction in the ECC condition than in the ISO condition, and residual force enhancement. First, joint torque at the onset of concentric contraction was larger in the ECC condition than in the ISO condition due to the type of preactivation. An eccentric contraction was conducted in the ECC condition, while an isometric contraction was conducted in the ISO condition. Because an eccentric contraction can produce a larger muscle force than an isometric contraction due to the force-velocity relationship [19, 20], the joint torque at the onset of concentric contraction should be higher in the ECC condition than in the ISO condition. This joint torque difference should affect to the joint torque, especially in the early phase of subsequent concentric contraction. However, joint torque in the ECC condition seemed to be higher at 105 degrees than that in the ISO, although the influence of preactivation and a larger joint torque at the onset of concentric contraction should disappear due to a long contraction duration (over 200 ms). This difference can be explained by the second reason, that is, residual force enhancement. Muscle force was enhanced temporarily after conducting an eccentric contraction [7, 8]. Previous studies have suggested that this effect (i.e., residual force enhancement) also contributes to torque enhancement by SSC [11, 21]. This effect can explain the increase in torque enhancement in the latter phase of concentric contraction in the ECC condition, which cannot be explained by preactivation. However, residual force enhancement is prominent in the descending limb and negligible in the ascending limb in terms of the force-length relationship [22, 23]. In the case of the physiological range of motion, some muscles work only in the ascending limb [24]. Considering a previous study that examined the working range in knee extensors [12], the knee extensors can partly work in the descending limb, so residual force enhancement can occur. To clarify this point, the working range of the sarcomere during physiological motion should be recorded and analyzed in the future.

Because fascicle behavior may contribute to torque enhancement in the ISO and ECC conditions, the pennation angle and fascicle length were measured in this study. Since the pennation angle was not different among the conditions, this factor cannot explain the observed differences in torque. Similarly, no significant difference in the fascicle length was observed among the conditions. However, because the fascicle length at 85 degrees seemed longer in the CON condition than in the others conditions (ISO and ECC), additional analyses were conducted. We calculated the magnitude of fascicle shortening obtained from 85 to 105 degrees (full range), 85 to 95 degrees (the first half), and 95 to 105 degrees (the latter half). As a result, the magnitude of fascicle shortening in full range was significantly larger in the CON condition than in the ISO and ECC conditions (Fig 7a). This difference was caused by the first half, not by the latter half (Fig 7b and 7c), of the concentric contraction phase. This result indicates that the observed difference in the magnitude of fascicle shortening would be caused by a slightly longer (but not significantly different) fascicle length at the onset of concentric contraction. Because the duration of shortening was identical among the conditions, the difference in the magnitude of fascicle shortening can be interpreted as the difference in the shortening velocity of the fascicle. As the shortening velocity of the fascicle increases, muscle force decreases due to the force-velocity relationship [25]. Thus, this fascicle behavior would also contribute to torque enhancement in the ISO and the ECC conditions. Generally, the magnitude of fascicle shortening at a given joint angle (i.e., at a given muscle-tendon complex length) has been considered to correspond to the magnitude of tendon elongation [4, 10, 26]. However, because the magnitude of the joint torque was about 25% of the maximal intensity (under 40 Nm) in our study, it is difficult to consider that substantial tendon elongation occurred. Thus, the observed fascicle shortening may be associated with the eliminating the slack of the muscle-tendon complex during the preactivation phase, which leads to a decrease in the magnitude of fascicle shortening in the subsequent concentric contraction phase.

The current study adopted electrically-evoked contractions, not voluntary contractions, to eliminate the influence of stretch reflex. Because electrically-evoked contractions are painful, especially in the maximal intensity contractions, a low intensity contraction (25% of the maximal intensity) was adopted. If the contraction intensity was higher than 25%, the result may be different. First, the influence of preactivation should be different. If the contraction intensity is higher than 25% in the preactivation phase, joint toque at the onset of concentric contraction is large. As a result, in the case of preactivation with a higher intensity condition, the joint torque should be larger in the early phase of concentric contraction. On the other hand, in the condition without preactivation, joint torque in the early phase should be small irrespective of the contraction intensity because the time to develop joint torque is insufficient. Thus, the difference in joint torque in the early phase of concentric contraction should be larger when the contraction intensity is higher. As a result, the influence of preactivation should become more evident. Second, the magnitude of tendon elongation may increase as the contraction intensity increases. In the present study, substantial tendon elongation would not be induced due to a low joint torque. However, in the condition where a larger joint torque is produced, substantial tendon elongation occurs, and consequently, tendon elongation may contribute to larger mechanical work during the subsequent concentric contraction phase in SSC. To clarify this point, changes in the tendon length should be measured at various contraction intensities. The tendon length changes should be measured directly not indirectly (i.e., subtracting the measured fascicle length changes from the estimated muscle-tendon complex length changes) because this indirect measurement cannot consider the fascicle shortening induced by eliminating slack.

In conclusion, torque enhancement by SSC in knee extensors would be mainly caused by preactivation. This speculation is supported by the findings that substantial torque enhancement occurred even without eccentric preactivation (ISO condition), and that the magnitude of torque enhancement with eccentric preactivation (ECC condition) was almost similar to that without eccentric preactivation (ISO condition). A slight but significant difference in torque enhancement would be caused by the difference in the torque at the onset of concentric contraction and residual force enhancement. In addition, fascicle behavior, that is, fascicle shortening during the preactivation phase that led to a decrease in the magnitude of fascicle shortening during the subsequent concentric contraction phase would also contribute to torque enhancement by SSC in knee extensors.
